# Supplementary material for: Building and Sustaining Scholarly Communities in Health Professions Education: Lessons from U.S. Academies
Source: Perspect Med Educ. 2026 Aug 25;15(1):675–86. doi: 10.5334/pme.3131 (PMC13523800; doi:10.5334/pme.3131)
Supplement: Supplementary file 1 [file pme-15-1-3131-s1.pdf]

Building and Sustaining Scholarly Communities in Health Professions Education
Interview Guide

Purpose of the Study

The purpose of this project is to identify best practices in health professions education research that will contribute to establishing a sustainable scholarly community of faculty at the George Washington University School of Medicine and Health Sciences. Through semi-structured interviews with academy leaders, this study aims to understand effective strategies in faculty development, mentorship, collaborative research, and program sustainability.

Interview Guide:

**Note:* The following dimensions of the Community of Practice framework are mapped to the questions below: Domain (D), Community (C), and Practice (P).

1. About You and Your Programs:

First, I'd like to learn more about you and your role:

- What are your roles and responsibilities in your medical school? (contextual)
- How long have you been with your institution and in this role? (contextual)
- How did you end up in this role? (contextual)

Let's talk now about any programs your school has that are designed to develop, sustain, and grow educational scholarship and research among your FACULTY:

- What programs do you have? (D, C, P)
    - For example, do you have an Academy of Education Scholars, an educational fellowship, or other programs?
- Please describe the general format of these programs: (C, P)
    - Probe regarding when started, requirements, number of faculty involved, format, face-to-face vs. virtual programming, etc.
    - How are faculty selected for this program? (C)

2. General Institutional Approach to Health Professions Education:

- How does your institution prioritize excellence in HPE scholarship and research? (D)
    - In other words, is it prioritized? And if so, in what ways? If not, why do you think it is not prioritized?

3. Best Practices in Educational Research:

- What practices, activities, and/or methodologies have proven most effective in helping faculty conduct high-impact educational research here? (P)
    - Can you provide a few examples?
- How do you support faculty in identifying relevant research questions that align with both institutional goals and current health education challenges? (D, P)
- What financial supports, if any, are faculty provided to engage in research, especially collaborative educational scholarship? (D)
- Are there specific training programs or resources that your faculty have found particularly valuable in developing research skills? (P)

4. Faculty Development and Support:

- What strategies does your institution use to build a culture that supports faculty development in educational research? (D, C)
- How do you balance research, teaching, and especially for clinicians, clinical expectations for faculty members, especially those interested in educational scholarship? (D, P)
- Lack of protected time appears to be one of our biggest challenges. Is that the case here too and, if so, how do you manage it? (D)
- What mentoring models and/or strategies do you implement to nurture emerging education scholars? (C, P)
- Finally, what sort of financial support does your institution provide to support faculty development in educational scholarship? (D)
    - In other words, what's your budget for this work?

5. Leadership in Education Scholarship:

- What qualities do you consider essential for someone leading an Academy of Education Scholars (or an educational fellowship or other structure designed to build educational research capacity among faculty members)? (C)
- How does your institution support its leaders? (D, C)
- How does your institution promote national and international collaboration in HPE research and scholarship? (C, P)

6. Challenges and Adaptations:

- What are some of the biggest challenges your institution has faced in advancing HPE scholarship, and how have you addressed them? (D, C, P)
- How do you manage things when money gets tight? (D)
    - In other words, my sense is that education research (and the support of it) is one of the first things to go when budgets tighten up. What's been your experience here, and how do you protect your efforts?
- Have you had to adapt your programs to meet evolving needs in healthcare and education? For example, the emergence of generative AI? (P)
    - If so, how have you done this?

7. Institutional Impact and Strategic Goals:

- How do you measure the impact of your educational scholarship on learners, your faculty (e.g., with respect to promotion and tenure), the institution, and even on the healthcare system writ large? (D, P)
    - How are these metrics gathered? Do you have admin support for this? (D)
- Do you share these metrics with the leadership and, if so, how? (D)
    - Do you write an annual report? If so, what does that look like and are you willing to share it with me after this interview?
- Do you have support for this effort in terms of writing, graphic design, etc.? (D, P)

8. Advice for Implementing Best Practices Elsewhere:

- If you could give one piece of advice to another institution trying to build a high-impact education scholars program, what would it be? (D, C, P)
- What common pitfalls have you observed, and how can they be avoided when establishing a similar program? (D, C, P)
